# UFBP1, a Key Component of the Ufm1 Conjugation System, Is Essential for Ufmylation-Mediated Regulation of Erythroid Development

**DOI:** 10.1371/journal.pgen.1005643

**Published:** 2015-11-06

**Authors:** Yafei Cai, Wenhu Pi, Satish Sivaprakasam, Xiaobin Zhu, Mingsheng Zhang, Jijun Chen, Levi Makala, Chunwan Lu, Jianchu Wu, Yong Teng, Betty Pace, Dorothy Tuan, Nagendra Singh, Honglin Li

**Affiliations:** 1 Department of Bioscience, College of Life Sciences, Anhui Normal University, Wuhu, Anhui, China; 2 Department of Biochemistry & Molecular Biology, Georgia Regents University, Augusta, Georgia, United States of America; 3 Department of Orthopedic Surgery, Zhongnan Hospital of Wuhan University, Wuhan, China; 4 Department of Oncology, Tongji Hospital, Tongji Medical College, Huazhong University of Science & Technology, Wuhan, China; 5 Department of Pediatrics, Georgia Regents University, Augusta, Georgia, United States of America; 6 Department of Periodontics, College of Dentistry, Cancer Center, University of Illinois at Chicago, Chicago, Illinois, United States of America; 7 Cancer Center, Georgia Regents University, Augusta, Georgia, United States of America; 8 The 10th People’s Hospital, Tongji University, Shanghai, China; Cincinnati Children's Hospital Medical Center, UNITED STATES

## Abstract

The Ufm1 conjugation system is an ubiquitin-like modification system that consists of Ufm1, Uba5 (E1), Ufc1 (E2), and less defined E3 ligase(s) and targets. The biological importance of this system is highlighted by its essential role in embryogenesis and erythroid development, but the underlying mechanism is poorly understood. UFBP1 (Ufm1 binding protein 1, also known as DDRGK1, Dashurin and C20orf116) is a putative Ufm1 target, yet its exact physiological function and impact of its ufmylation remain largely undefined. In this study, we report that UFBP1 is indispensable for embryonic development and hematopoiesis. While germ-line deletion of *UFBP1* caused defective erythroid development and embryonic lethality, somatic ablation of *UFBP1* impaired adult hematopoiesis, resulting in pancytopenia and animal death. At the cellular level, *UFBP1* deficiency led to elevated ER (endoplasmic reticulum) stress and activation of unfolded protein response (UPR), and consequently cell death of hematopoietic stem/progenitor cells. In addition, loss of UFBP1 suppressed expression of erythroid transcription factors GATA-1 and KLF1 and blocked erythroid differentiation from CFU-Es (colony forming unit-erythroid) to proerythroblasts. Interestingly, depletion of Uba5, a Ufm1 E1 enzyme, also caused elevation of ER stress and under-expression of erythroid transcription factors in erythroleukemia K562 cells. By contrast, knockdown of ASC1, a newly identified Ufm1 target that functions as a transcriptional co-activator of hormone receptors, led to down-regulation of erythroid transcription factors, but did not elevate basal ER stress. Furthermore, we found that ASC1 was associated with the promoters of *GATA-1* and *Klf1* in a UFBP1-dependent manner. Taken together, our findings suggest that UFBP1, along with ASC1 and other ufmylation components, play pleiotropic roles in regulation of hematopoietic cell survival and differentiation via modulating ER homeostasis and erythroid lineage-specific gene expression. Modulating the activity of this novel ubiquitin-like system may represent a novel approach to treat blood-related diseases such as anemia.

## Author Summary

Protein modification by Ubiquitin (Ub) and Ubiquitin-like proteins (Ubl) plays pivotal roles in a wide range of cellular functions and signaling pathways. The Ufm1 conjugation system is a novel ubiquitin-like system, yet its biological functions and working mechanism remains poorly understood. UFBP1 is a putative Ufm1 target that has been implicated in several signaling pathways but little is known regarding its *in vivo* function. In this report, by using multiple knockout mouse models, we demonstrate that UFBP1 is essential for murine development and blood cell development. While germ-line deletion of *UFBP1* caused defective red blood cell development and embryonic lethality, somatic ablation of *UFBP1* impaired production of mature red blood cells and other types of hematopoietic cells. We found that depletion of UFBP1 led to elevated stress in the endoplasmic reticulum that in turn caused cell death of hematopoietic stem cells. Furthermore, UFBP1 deficiency diminished expression of key transcription factors essential for red blood cell development. Taken together, our study provides strong genetic evidence for the essential role of UFBP1 as well as other components of the Ufm1 system in hematopoietic development. Therefore, the ufmylation pathway may represent a novel therapeutic target in treatment of blood diseases.

## Introduction

The Ufm1 (Ubiquitin-fold modifier 1) conjugation system is a novel ubiquitin-like (Ubl) modification system that mediates protein modification by a small protein modifier Ufm1 [1]. Ufmylation is catalyzed by a series of enzymes, consisting of Ufm1-activating E1 enzyme (Uba5), Ufm1-conjugating E2 enzyme (Ufc1), and a newly identified E3 ligase Ufl1 [1–3]. The genetic study using *Uba5* knockout mice demonstrates that Uba5 is essential for embryogenesis and erythroid development, highlighting the importance of this system in animal development [4]. Yet, its downstream effector(s) and molecular mechanism remain poorly understood.

UFBP1 (Ufm1 binding protein 1 with a PCI domain, also known as C20orf116, Dashurin, and DDRGK1) is a highly conserved protein whose orthologues are found in metazoan and plants but not in yeast, indicating its important role in multicellular organisms [2, 5–7]. It is ubiquitously expressed in multiple tissues and organs with high- level expression in secretory cells [7]. Human UFBP1 contains 314 amino acid residues with a putative signal peptide at its N-terminus that anchors it to the endoplasmic reticulum (ER) [2, 6]. It also contains a partial PCI domain that is frequently found in the subunits of the proteasome, COP9 signalosome and translation initiation factors. UFBP1 is found to associate with two other proteins namely C53 (also designated as LZAP, Cdk5rap3) and Ufl1 (also known as KIAA0776, NLBP, RCAD and Maxer) [2, 6]. Interestingly, Tatsumi *et al* found that UFBP1 was a Ufm1 target and its ufmylation at the highly conserved residue Lys267 was promoted by the Ufm1 E3 ligase Ufl1 [2]. Moreover, Yoo *et al* have recently demonstrated that the ufmylated form of UFBP1 is a co-component of Ufl1 E3 ligase that promotes ufmylation of nuclear receptor co-activator ASC1, an event that is crucial for estrogen receptor signaling and breast cancer development [8]. Nonetheless, little is known concerning the *in vivo* function of UFBP1 and the functional impact of its ufmylation.

In this study, we report the essential role of UFBP1 in murine development and hematopoiesis. Ablation of UFBP1 leads to impaired embryogenesis and defective hematopoiesis. Our results strongly suggest that UFBP1 functions as a key player in the ufmylation pathway that is crucial for cellular homeostasis and erythroid development.

## Results

### UFBP1 is essential for embryonic development

**Fig 1:**
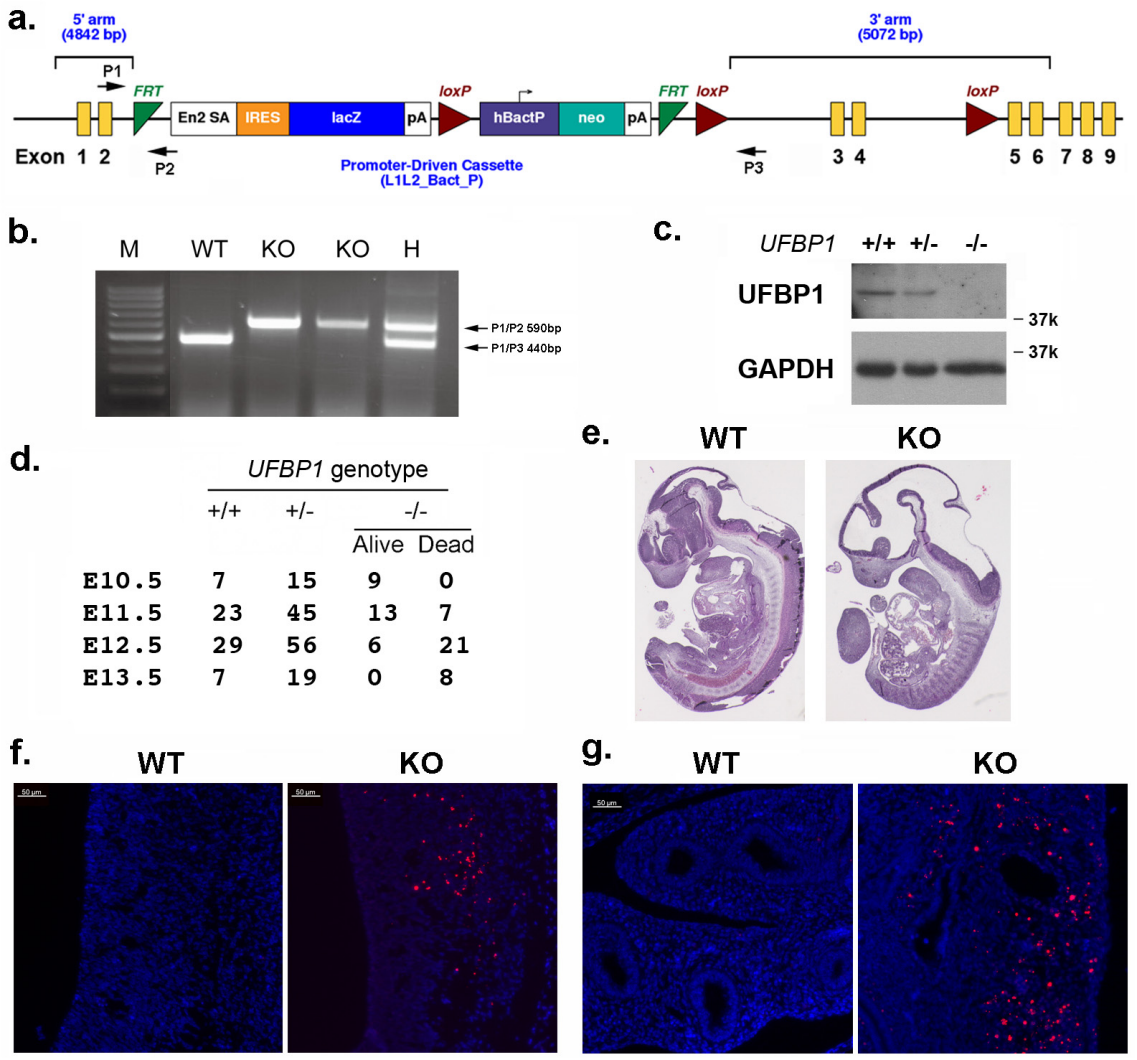
UFBP1 is essential for embryonic development. **a**. The targeting vector of *UFBP1* allele. **b.** PCR-based genotyping of *UFBP1* allele with the gene trap cassette. **c**. Immunoblotting of UFBP1 protein in wild-type (WT) and KO embryos. **d**. Embryonic lethality of *UFBP1* null embryos. **e**. Hematoxylin & eosin (H & E) staining of representative WT and KO embryos (E11.5)**. f.** TUNEL staining of the bottom of the fourth ventricle of embryonic brain in E11.5 embryos. **g.** TUNEL staining of lumen of duodenum in E11.5 embryos. Scale bar: 50 μm.

In an attempt to understand UFBP1’s *in vivo* function, we generated *UFBP1* knockout mice. The murine *UFBP1* gene is located in chromosome 2 and consists of 9 exons (Fig 1A). A gene trap cassette flanked by two FRT sites was inserted into the intron between exon 2 and 3 and followed by floxed exons 3 and 4, thereby creating a *UFBP1* ^Trap-F^ allele (Fig 1A). Insertion of the gene trap was confirmed by genomic PCR (Fig 1B). Disruption of *UFBP1* expression was confirmed by the complete absence of UFBP1 protein in the embryos with homozygous trapped alleles (Fig 1C). Therefore, the mice with homozygous trapped alleles (*UFBP1* ^Trap-F/Trap-F^) were designated as *UFBP1* ^*-/-*^ or *UFBP1* KO mice.

**Fig 2:**
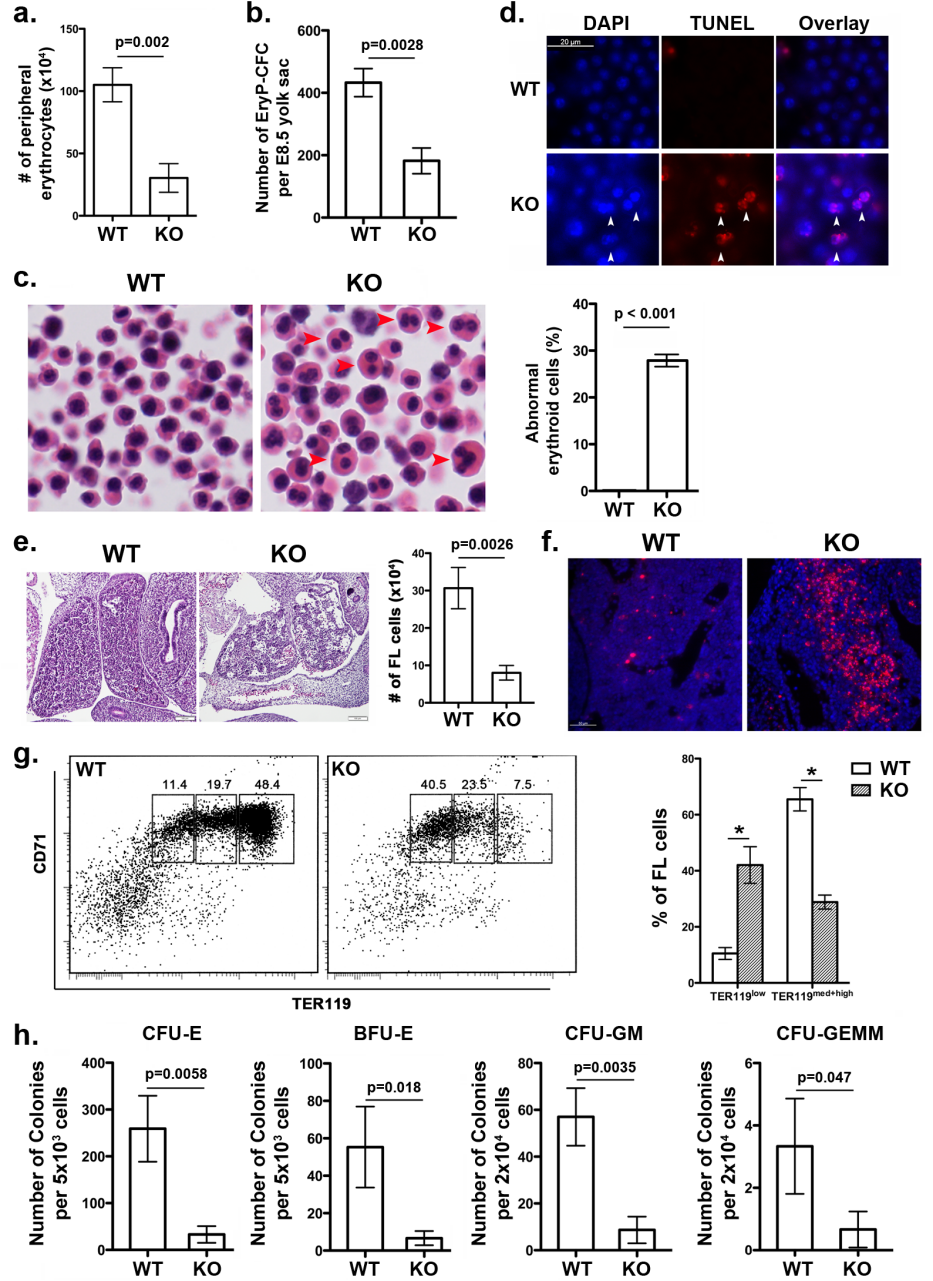
*UFBP1* deficiency impairs embryonic erythroid development. **a**. The number of erythrocytes in peripheral blood of WT and KO E11.5 embryos. Blood cells were collected from suspension solution during separation of yolk sac from embryos and manually counted. Data are presented as means ± SD (n = 3). **b.** The number of EryP-CFC in E8.5 yolk sac. Data are presented as means ± SD (n = 3). **c.** Abnormal multi-nucleated erythroid cells in E11.5 embryos. Multi-nucleated erythrocytes are marked by red arrowheads in H & E stained sections. Data are presented as means ± SD (n = 3). **d.** Representative photographs of TUNEL staining of circulating erythrocytes in E11.5 embryos. **e.** H & E staining and number of fetal liver cells in E11.5 embryos. The number of fetal liver cells was scored by counting total cells from dissected fetal livers. Data are presented as means ± SD (n = 3). **f.** Representative photographs of TUNEL staining of fetal liver sections (E11.5). **g.** Representative flow cytometry analysis of fetal liver cells from E11.5 embryos. Fetal liver cells were freshly isolated from E11.5 embryos and subjected to flow cytometry analysis of CD71 and TER119 markers. Percentages of each population of cells are presented as means ± SD (n = 3). **h.** CFU-Es, BFU-Es, CFU-GMs, and CFU-GEMMs in E11.5 fetal livers. Data are presented as means ± SD (n = 3).

Among 158 adult mice, we failed to obtain *UFBP1* ^*-/-*^ animals while 51 *UFBP1* ^+/+^ and 107 *UFBP1* ^+/-^ mice were born healthy and appeared normal, indicating a possible embryonic lethality caused by loss of UFBP1. By analyzing the embryos of timed-pregnant mice, we found that most *UFBP1* ^*-/-*^ embryos died at E12.5 (Fig 1D), a phenotype which is similar to the ones of *Uba5* and *Ufl1* KO mice but slightly delayed compared to *Ufl1* KO mice [9]. E11.5 *UFBP1* ^*-/-*^ embryos appeared smaller and paler than their wild-type littermates (Fig 1E and S1 Fig). Extensive cell death (TUNEL staining) was observed in brain, liver and other tissues (Figs 1F, 1G and 2B). Taken together, our results suggest that UFBP1 is essential for proper embryogenesis and its deficiency leads to elevated cell death.

### UFBP1 deficiency impairs embryonic erythropoiesis

Ablation of either Uba5 or Ufl1 leads to defective embryonic erythropoiesis [4, 9]. Therefore, it is of great interest to determine if *UFBP1* deficiency also impacts on embryonic erythroid development. At E11.5, *UFBP1* KO embryos had fewer peripheral circulating erythrocytes compared to wild-type embryos (Fig 2A), while *UFBP1* deficient yolk sac (E8.5) contained a reduced number of primitive erythroid colony-forming cells (EryP-CFC) (Fig 2B). Additionally, there were a large number of abnormal multinucleated erythroid cells in *UFBP1* ^*-/-*^ embryos, a phenotype reminiscent of *Uba5* and *Ufl1* null embryos (Fig 2C) [4, 9]. Some multi-nucleated erythroid cells were TUNEL-positive, indicating occurrence of cell death of these cells (Fig 2D). These results indicate an impairment of primitive erythropoiesis in *UFBP1* deficient embryos. We also examined definitive erythropoiesis in fetal livers. *UFBP1* KO embryos (E11.5) had small and dis-organized fetal livers with less cellularity (Fig 2E) and increased cell death (Fig 2F). Flow cytometry analysis of E11.5 fetal liver cells showed that *UFBP1* deficiency significantly blocked differentiation of TER119^low^ cells into TER119^med+high^ cells (proerythroblasts and basophilic erythroblasts) cells (Fig 2G). Furthermore, the numbers of erythroid and myeloid progenitor cells, including CFU-Es (colony-forming units-erythrocytes), BFU-Es (erythroid burst-forming units), CFU-GM (colony-forming units- granulocyte/macrophage) and CFU-GEMM (colony-forming units- granulocyte/erythrocyte/macrophage/megakaryocyte), were significantly lower in *UFBP1* ^*-/-*^ fetal livers (E11.5) (Fig 2H). Taken together, our results suggest that UFBP1 plays an indispensable role in both primitive and definitive erythropoiesis during embryonic development.

### Ablation of UFBP1 causes severe pancytopenia in adult mice

**Fig 3:**
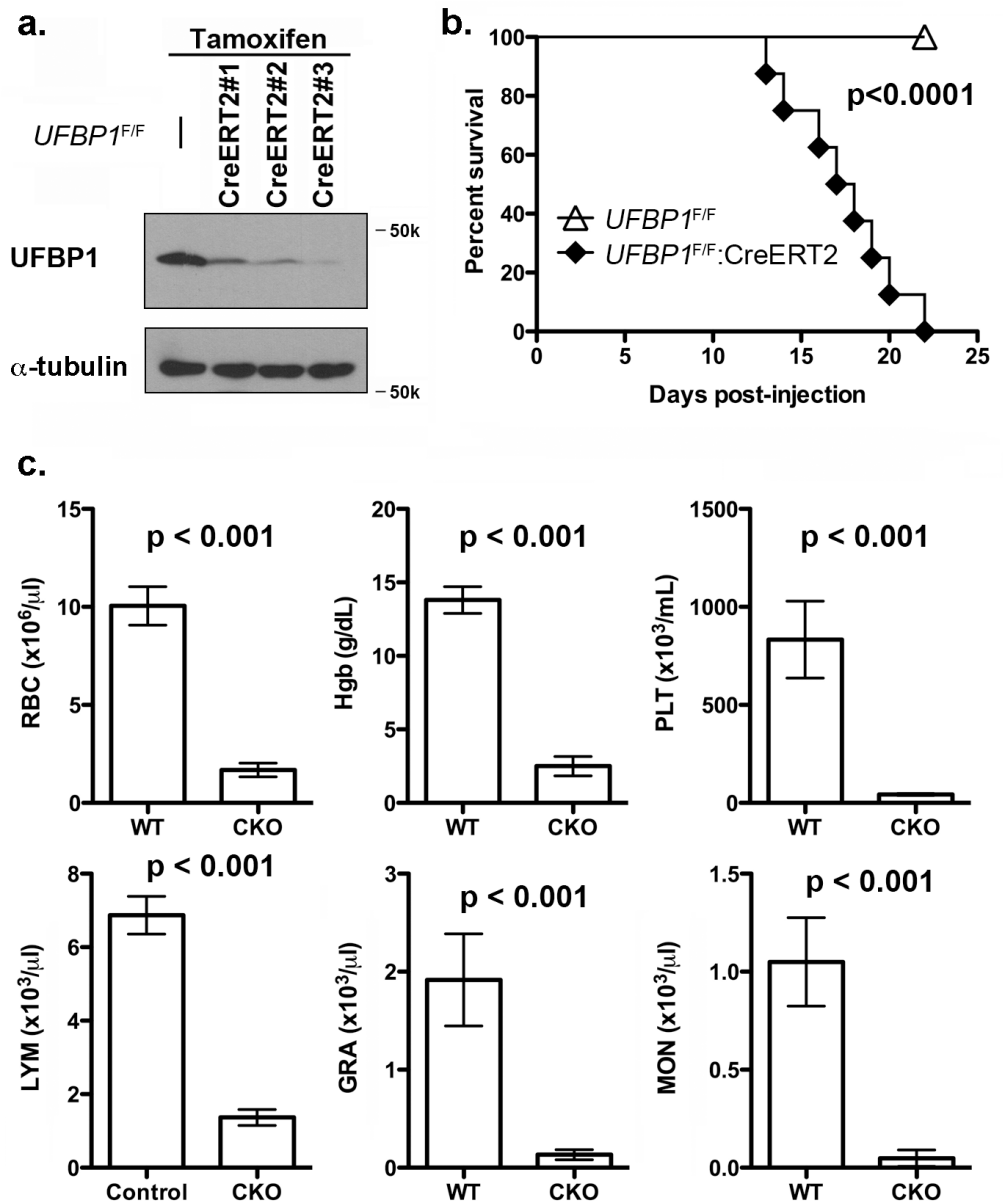
Loss of UFBP1 in adult mice results in severe pancytopenia and animal death. **a**. Confirmation of UFBP1 depletion in bone marrow cells of TAM-injected CKO mice. Floxed mice were IP injected with tamoxifen according to a standard protocol. BM cells were collected and subject to immunoblotting of UFBP1. **b.** Survival curve of *UFBP1* deficient mice after TAM injection, p < 0.001 (n = 12 each group). **c**. The result of CBC counts. Data are presented as means ± SD. The blood was drawn from control (*UFBP1* ^F/F^) (n = 5) and *UFBP1* deficient mice (*UFBP1* ^F/F^:CreERT2) (n = 5) when *UFBP1* deficient mice became moribund and/or lost 20% of body weight after TAM injection (between 2–3 weeks post-TAM treatment). Blood samples were subjected to CBC analysis. The mice used in this experiment were ~ 8-week old male mice. RBC: red blood cell; Hgb: hemoglobin; PLT: platelet; LYM: lymphocyte; GRA: granulocyte; MON: monocyte.

To investigate the *in vivo* role of UFBP1 in adult erythropoiesis, we generated inducible conditional KO (CKO) mice of *UFBP1* via a two-step procedure: 1) removing the gene trap cassette by crossing *UFBP1* ^Trap-F/+^ mice with FLPo deleter mice; and 2) crossing *UFBP1* floxed mice with ROSA26-CreERT2 mice. As shown in Fig 3A, endogenous UFBP1 in bone marrow (BM) cells was depleted in tamoxifen (TAM)-treated *UFBP1* ^F/F^:ROSA26-CreERT2 mice (designated as *UFBP1* ^F/F^:CreERT2 hereafter) but not in *UFBP1* ^F/F^ mice (Fig 3A). Interestingly, UFBP1-depleted mice suffered substantial loss of body weight and died around 3 weeks after initial TAM treatment, while *RCAD* ^F/F^ mice were fairly normal (Fig 3B). In the hematopoietic system, *UFBP1* deficient mice exhibited severe pancytopenia (Fig 3C). The major indices for erythrocytes, including RBC (red blood cell) count and Hgb (hemoglobin) content, were significantly lower in *UFBP1* deficient mice. Moreover, the counts for lymphocytes, granulocytes, monocytes, as well as platelet counts were also dramatically decreased in *UFBP1* deficient mice (Fig 3C). This result strongly indicates an essential role of UFBP1 in adult hematopoiesis.

### Loss of UFBP1 impairs erythroid development at multiple stages

**Fig 4:**
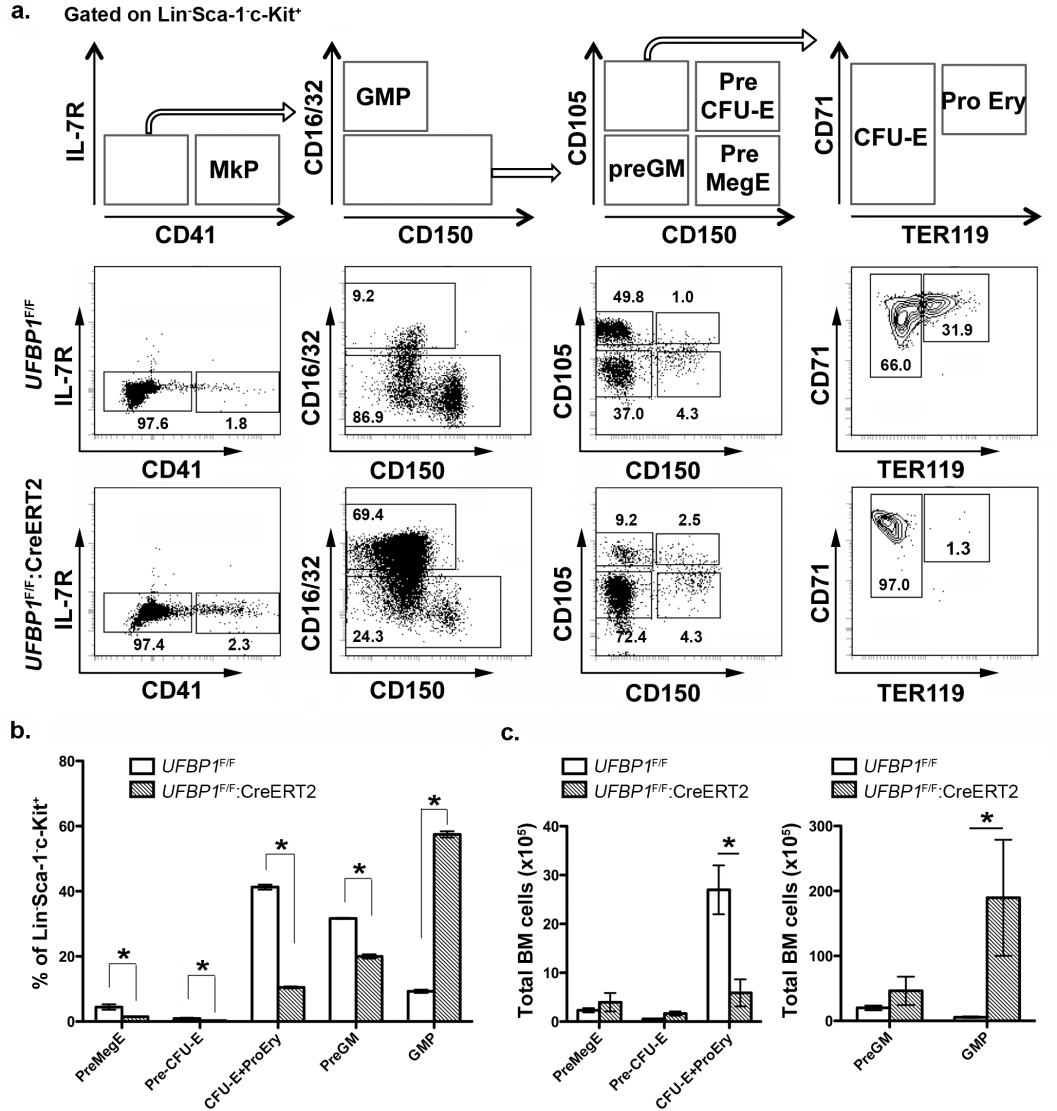
*UFBP1* deficiency impairs lineage development of erythroid progenitors. **a**. Flow cytometry analysis of erythroid and myeloid lineages in control and *UFBP1* deficient mice after TAM injection. BM cells were collected from TAM-injected control (n = 4) and floxed (n = 4) male mice when *UFBP1* deficient mice exhibited significant weight loss and became moribund. The following markers were used for flow analysis: BV510-Sca-1, APC780-c-Kit, PE-Cy7-CD150, Alexa 700-CD16/32, PE-IL-7R, APC-CD41, BV650-CD105, FITC-CD71, BV421-TER119, and lineage markers including PerCP-Cy5.5-conjugated CD4, CD8, CD3, CD5, Gr-1, CD11b, CD19 and B220. **b.** The percentages of each lineage in L^-^S^-^K^+^ cells. *p <0.01, and **p <0.05 (n = 4). MkP: megakaryocyte progenitor; GMP: granulocyte macrophage progenitor; Pre GM: granulocyte macrophage precursor; Pre MegE: megakaryocyte erythroid precursor; Pre CFU-E: CFU-E precursor; CFU-E: erythroid colony-forming unit; Pro Ery: proerythroblast. **c**. The absolute cell numbers of each lineage in control and UFBP1-depleted BMs. UFBP1 depletion led to reduction of total BM cells (1 x 10^8^ cells in control versus 5 x 10^7^ in *UFBP1* deficient mice). *p <0.01 (n = 4).

To further define the role of UFBP1 in adult erythropoiesis, we analyzed erythroid development in TAM-treated *UFBP1* CKO mice according to Pronk *et al* [10]. The myeloerythroid (Lin^-^Sca-1^-^c-kit^+^IL-7R^-^) fraction of BM cells was subject to flow cytometry analysis using indicated markers (Fig 4A). As shown in Fig 4A, significant changes in myeloid and erythroid lineages were observed in *UFBP1* deficient BM cells. The percentages of all types of erythroid progenitors, including Pre MegE (4.40 ± 0.81% vs 1.42 ± 0.16%), Pre CFU-E (0.89 ± 0.22% vs 0.25 ± 0.07%), and CFU-E plus proerythroblasts (41.23 ± 0.78% vs 10.43 ± 0.32%), were significantly decreased in TAM-treated *UFBP1* ^F/F^:CreERT2 mice compared to *UFBP1* ^F/F^ mice (Fig 4A and 4B). Moreover, differentiation from CFU-Es (TER119^low^) to proerythroblasts (TER119^high^) was almost completely blocked by loss of UFBP1 (Fig 4A, the last panel). By contrast, the percentage of GMPs (9.24 ± 0.51% vs 57.40 ± 0.95%) was substantially increased in *UFBP1* deficient BM, while the percentage of Pre GMs (31.60 ± 0.17% vs 19.9 ± 0.64%) was modestly decreased (Fig 4A and 4B). The total cell number of erythroid progenitors (CFU-Es + proerythroblasts) was also reduced in *UFBP1* deficient BM, suggesting that UFBP1 plays a crucial role in development of erythroid lineage. Interestingly, although the number of GMPs in the UFBP1-deleted BM was dramatically expanded (Fig 4C), the number of mature monocytes/granulocytes in peripheral blood was decreased (Fig 3C), indicating that UFBP1 may also have a role in development of myeloid lineages.

### UFBP1 deficiency impairs hematopoietic stem cell (HSC) function in a cell-autonomous manner

**Fig 5:**
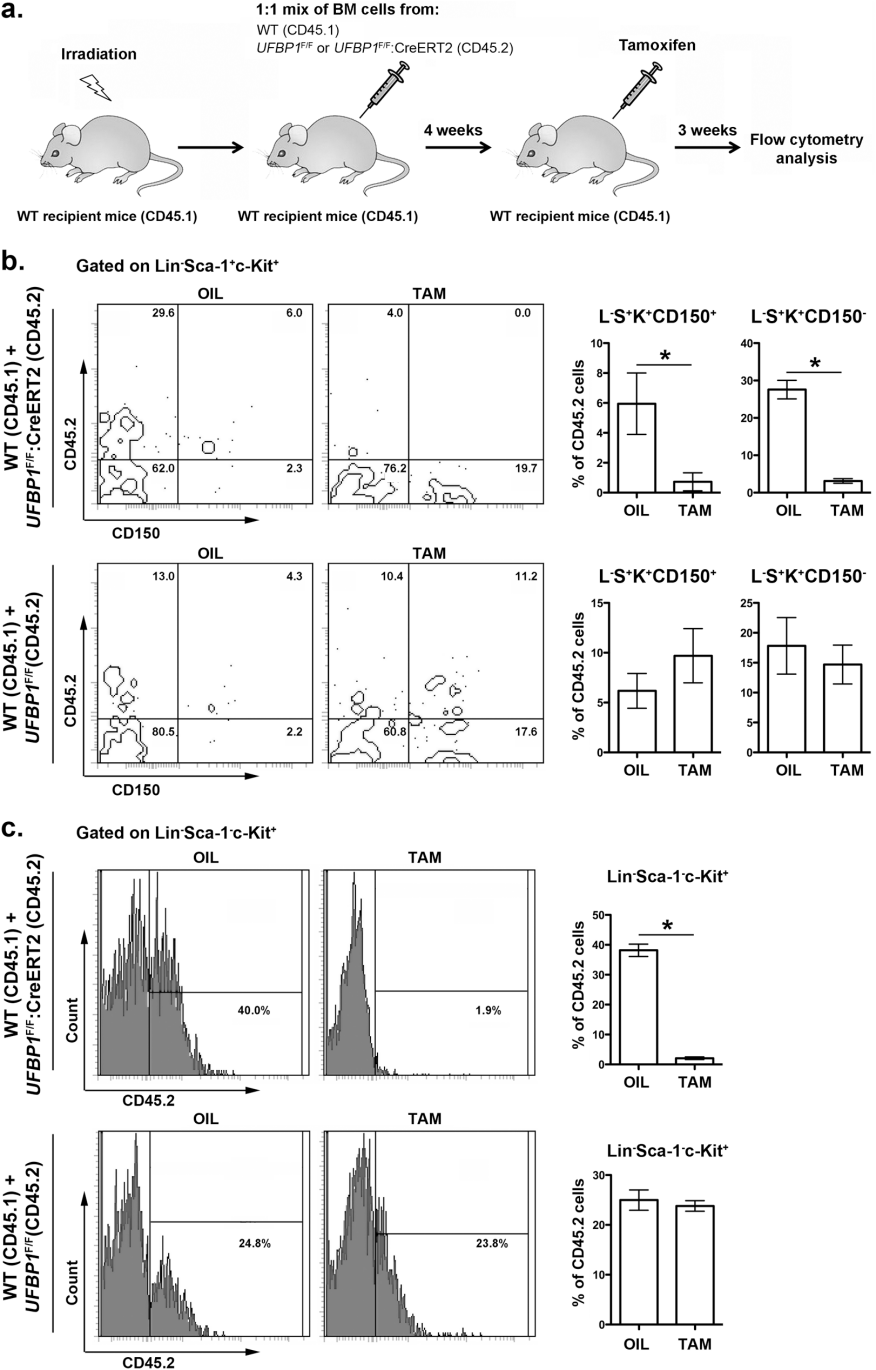
UFBP1 is essential for HSC function. **a.** Experimental scheme for competitive repopulation assay. **b.** Contribution of *UFBP1* deficient cells (CD45.2) to long-term HSCs (L^-^S^+^K^+^CD150^+^) and multipotent progenitors (L^-^S^+^K^+^CD150^-^). * p < 0.001 (n = 5). **c.** Contribution of *UFBP1* deficient cells (CD45.2) to oligopotent progenitor cells (L^-^S^-^K^+^). * p < 0.001 (n = 5). Data are presented as means ± SD. The unfractionated BM cells from either *UFBP1* ^F/F^ or *UFBP1* ^F/F^:CreERT2 (CD45.2) were mixed with wild-type (CD45.1) BM cells at a 1:1 ratio, and co-transplanted into lethally irradiated recipient CD45.1 mice. Four weeks after transplantation, the mice were treated with either oil or TAM. Three weeks after initiation of the treatment, the BM cells were isolated and subjected to flow analysis using indicated markers.

In addition to impaired erythropoiesis, *UFBP1* deficient mice also exhibited cytopenia of other types of hematopoietic cells (Fig 3C), indicating a possible loss of HSC function. In line with this hypothesis, *UFBP1* deficient BMs failed to rescue lethally irradiated recipient mice (S2 Fig). To further assess the cell intrinsic role of UFBP1 in HSC function, we performed competitive repopulation assays. Unfractionated BM cells from either *UFBP1* ^F/F^ or *UFBP1* ^F/F^:CreERT2 (CD45.2) were mixed with wild-type (CD45.1) BM cells at a 1:1 ratio, and co-transplanted into lethally irradiated recipient CD45.1 mice (Fig 5A). Four weeks after transplantation, mice were treated with either oil or tamoxifen. Three weeks after initiation of the treatment, we examined the contribution of donor cells (CD45.2) to HSCs and other progenitor cells in BM. As shown in S3 Fig, the contribution of *UFBP1* deficient (*UFBP1* ^F/F^:CreERT2) cells to Lin^-^Sca-1^+^c-Kit^+^ (LSK) population was substantially diminished after TAM treatment comparing to oil treatment (32.78 ± 1.79% vs 3.35 ± 0.28%). By contrast, TAM treatment yielded no effect on the contribution of *UFBP1* ^F/F^ cells (S3 Fig). Further analysis using CD150 marker showed that the contribution of *UFBP1* deficient cells to both long-term HSCs (L^-^S^+^K^+^CD150^+^) (5.95 ± 1.02% vs 0.73 + 0.30%) and multipotent progenitors (L^-^S^+^K^+^CD150^-^) (27.58 ± 1.24% vs 3.13 ± 0.31%) was significantly reduced (Fig 5B). Similarly, oligopotent progenitors (L^-^S^-^K^+^) (38.18 ± 1.08% vs 2.08 ± 0.21%) contained much lower level of *UFBP1* deficient cells (Fig 5B). The result of competitive repopulation assays strongly suggests that UFBP1 is essential for HSC function in a cell-autonomous manner.

### UFBP1 depletion activates the UPR and cell death program in LSK cells

**Fig 6:**
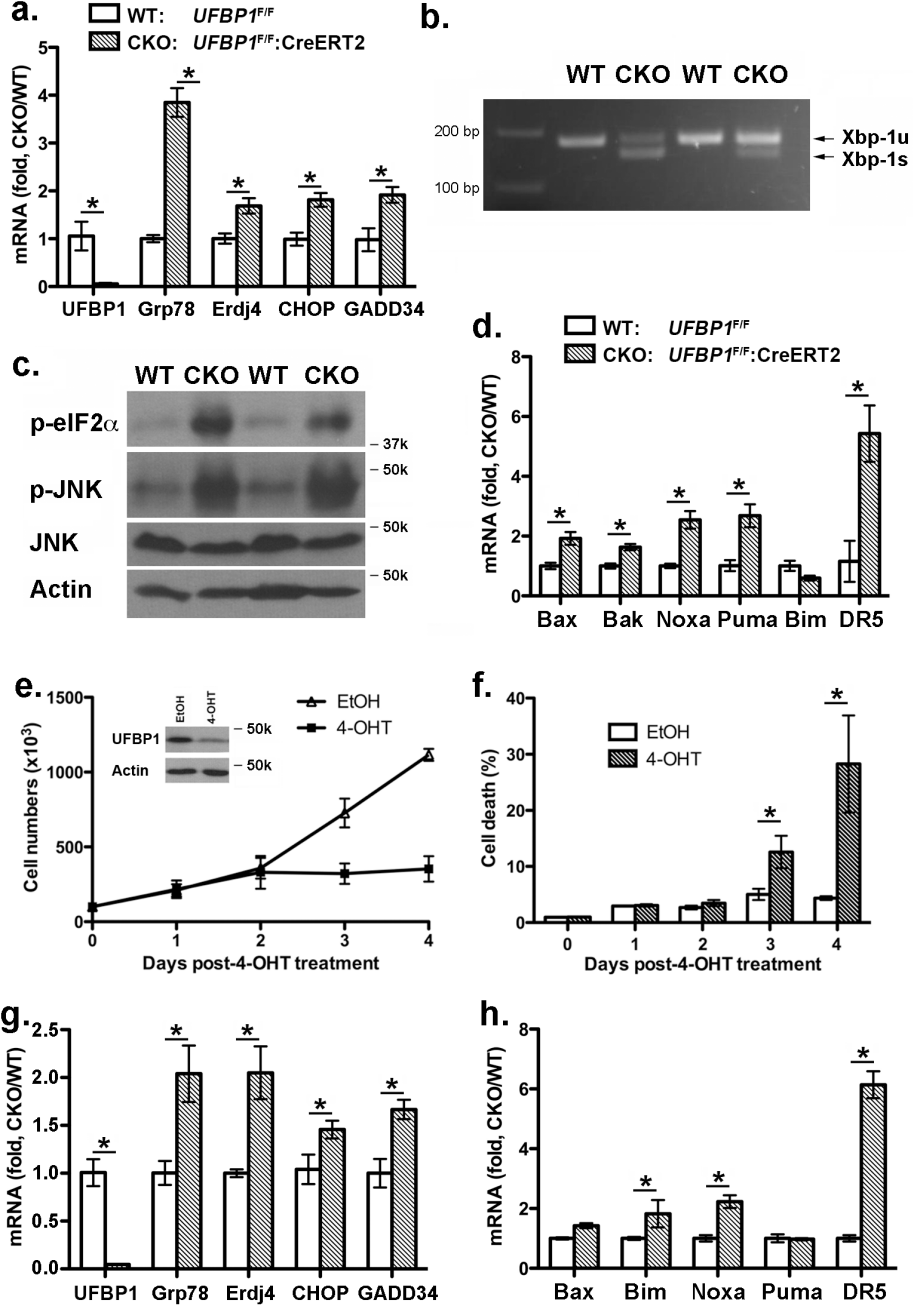
Loss of UFBP1 activates the UPR and cell death program. **a**. Up-regulation of Grp78, ERdj4 and CHOP in *UFBP1* deficient BM cells. Total RNAs were purified from BM cells and subject to quantitative RT-PCR analysis (normalized to β-actin gene). TAM-injected *UFBP1* ^F/F^mice were used as WT control mice, while TAM-injected *UFBP1* ^F/F^:CreERT2 mice were designated as CKO mice. The ratio of CKO to WT was presented. * p < 0.01 (n = 3). Data are presented as means ± SD. **b**. *Xbp-1* mRNA splicing in *UFBP1* deficient BM cells. Total RNAs were isolated from BM cells of control and CKO mice, and subjected to *Xbp-1* mRNA splicing assay. **c.** Elevation of phosphorylation of eIF2α and JNK in *UFBP1* deficient cells. Total cell lysates of BM cells were subjected to immunoblotting of indicated antibodies. **d.** Up-regulation of cell death genes in *UFBP1* deficient BM cells. Total RNAs were purified from BM cells and subject to quantitative RT-PCR analysis (normalized to β-actin gene). The ratio of CKO to WT was presented. * p < 0.01 (n = 3). **e.** Proliferation of wild-type and *UFBP1* deficient LSK cells. LSK cells were sorted from BM of *UFBP1* ^F/F^:CreERT2 mice, and cultured in the absence or presence of 4-OHT (1 μM) for indicated period of time. Cell numbers were manually scored. The experiment was performed three times independently. **f.** Cell death of *UFBP1* deficient HLSK cells. Cell death was elevated by DNA dye exclusion assay. **g.** Up-regulation of UPR genes in *UFBP1* deficient LSK cells. Total RNAs were purified from LSK cells and subject to quantitative RT-PCR analysis (normalized to β-actin gene). The ratio of CKO to WT was presented. * p < 0.01 (n = 3). **h.** Up-regulation of cell death genes in *UFBP1* deficient LSK cells. The ratio of CKO to WT was presented (normalized to β-actin gene). * p < 0.01 (n = 3).

UFBP1 and other components of the Ufm1 system play a cytoprotective role in ER stress-induced apoptosis [7, 11]. Therefore, we examined the effect of *UFBP1* deficiency on ER homeostasis. Expression of ER chaperones Grp78 and ERdj4 was up-regulated in *UFBP1* deficient BM cells (Fig 6A), and UFBP1 depletion induced *Xbp-1* mRNA splicing (Fig 6B) and elevation of phosphorylation of eIF2α and JNK (Fig 6C). These results indicate that *UFBP1* deficiency indeed led to elevated ER stress and activation of the UPR. Furthermore, a subset of apoptotic genes associated with ER stress, including Bax, Bak, Noxa, Puma and DR5, were up-regulated in *UFBP1* deficient cells (Fig 6D). In contrast to UFL1 deficient cells, however, we did not observe apparent inhibition of autophagic flux in UFBP1 deficient BM cells (S4 Fig). We also examined the effect of *UFBP1* depletion on LSK cells *in vitro*. Sorted *UFBP1* ^F/F^:CreERT2 LSK cells were treated with 4-hydroxytamoxifen (4-OHT) and cultured for indicated time. 4-OHT-induced depletion of UFBP1 led to a significant decrease of proliferation and increase of cell death of LSK cells (Fig 6E and 6F). Consistent with the *in vivo* result, the UPR and cell death genes were up-regulated in UFBP1-depleted LSK cells (Fig 6G and 6H). Collectively, our results suggest that UFBP1 plays an essential role in survival of LSK progenitor cells by maintaining ER homeostasis.

### Loss of ufmylation capacity leads to elevated ER stress and suppression of erythroid transcriptional program

**Fig 7:**
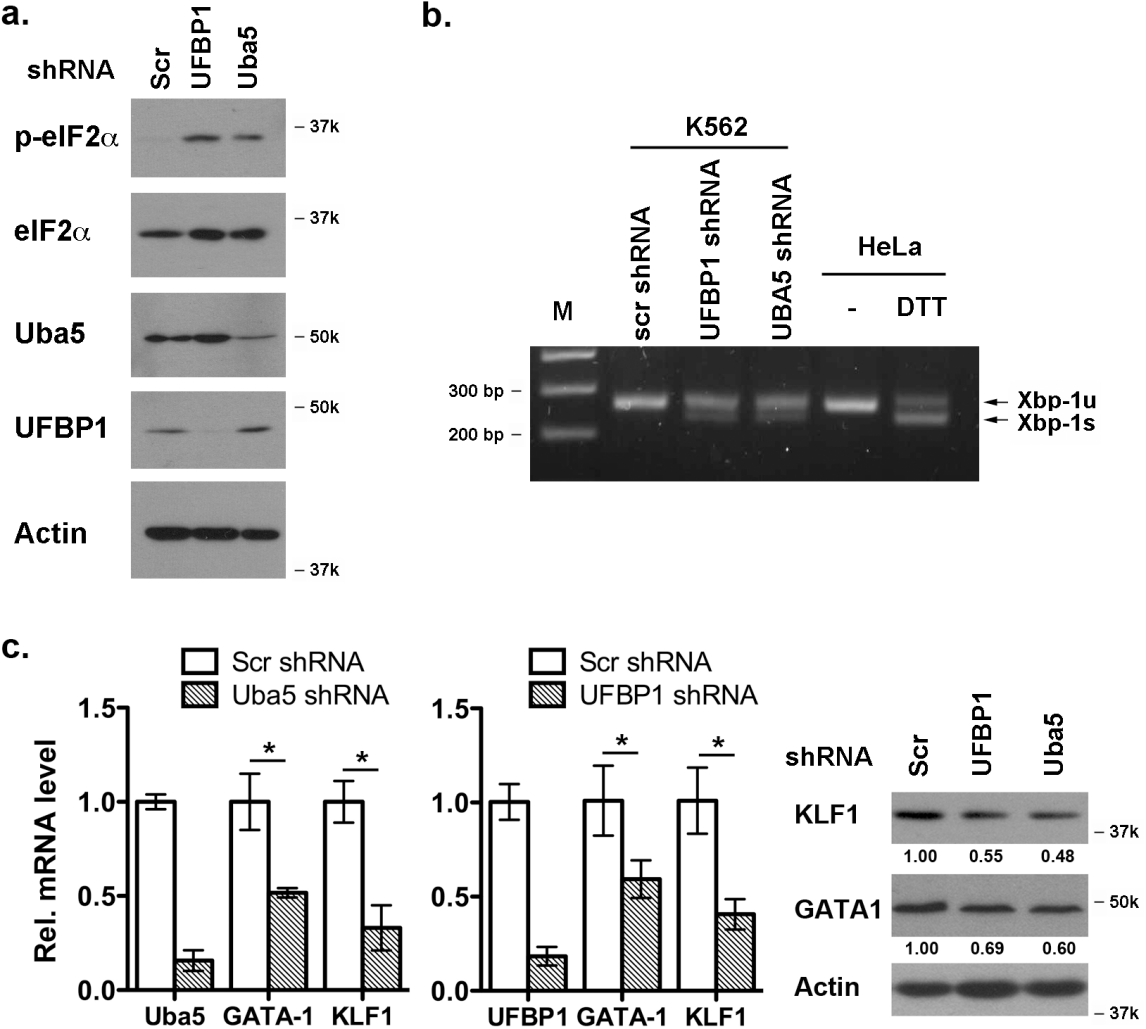
Depletion of Uba5 activates the UPR and suppresses expression of erythroid transcription factors in K562 cells. **a.** Elevation of phosphorylation of eIF2α in *Uba5*- and *UFBP1*-depleted K562 cells. **b**. *Xbp-1* mRNA splicing in *Uba5*- and *UFBP1*-depleted K562 cells. mRNA from DTT (dithiothreitol)-treated HeLa cells was used as a positive control. **c.** Under-expression of *GATA-1* and *Klf1* in *Uba5*- and *UFBP1*-depleted K562 cells. Total RNAs were purified from K562 cells and subject to quantitative RT-PCR analysis (normalized to β-actin gene). The data are presented as the relative level to the K562 cells treated with scrambled shRNA. * p < 0.01 (n = 3). Total cell lysates were subject to immunoblotting of indicated antibodies.

It has been shown that Uba5 is essential for embryonic erythropoiesis, but the underlying mechanism remains poorly understood [4]. We examined whether Uba5 exerts its function in similar cellular pathways in which UFBP1 is involved. As shown in Fig 7A and 7B, knockdown of either Uba5 or UFBP1 in erythroleukemia K562 cells caused the increase of eIF2α phosphorylation and Xbp-1 mRNA processing, indicating activation of the UPR in these knockdown cells. This result suggests that Uba5 is also involved in regulation of ER homeostasis. Furthermore, depletion of either Uba5 or UFBP1 down-regulated key erythroid transcription factors GATA-1 and KLF1 in K562 cells (Fig 7C), suggesting a lineage-specific role of ufmylation in erythropoiesis. Together, our results provide the first evidence suggesting that the Ufm1 system as a whole have pleiotropic roles in multiple cellular processes including maintenance of ER homeostasis and transcriptional regulation of erythroid development.

### Depletion of ASC1, a Ufm1 target, down-regulates the erythroid transcriptional program but does not elevate ER stress

**Fig 8:**
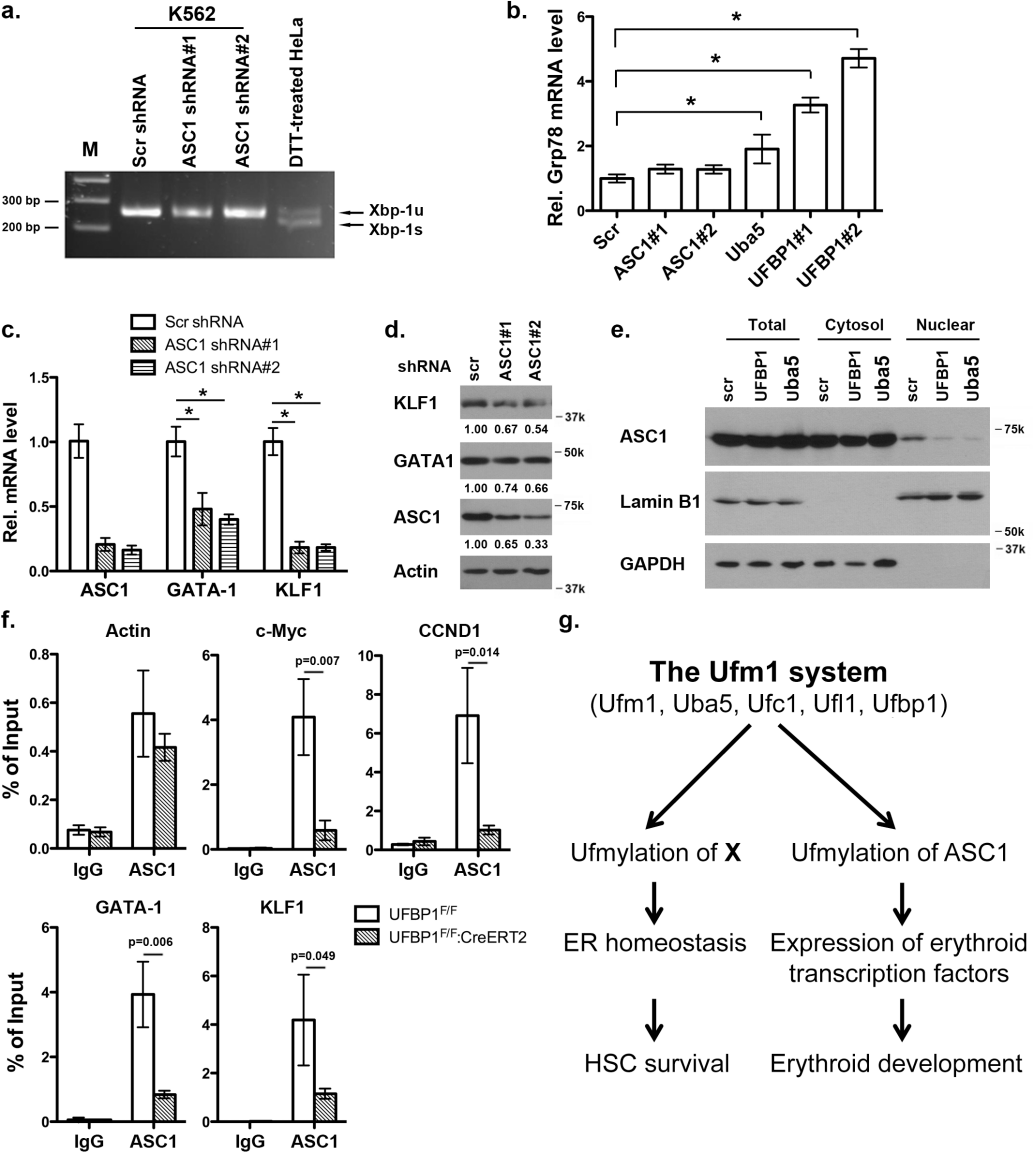
Knockdown of ASC1 down-regulates expression of erythroid transcription factors but does not activate the UPR. **a.** *Xbp-1* mRNA splicing in *ASC1*-depleted K562 cells. mRNA from DTT (dithiothreitol)-treated HeLa cells was used as a positive control. **b.** Expression of *Grp78* in K562 cells with knockdown of ASC1, Uba5 and UFBP1. The data are presented as the relative level to K562 cells treated with scrambled shRNA. * p < 0.01 (n = 3). **c.** Expression of *GATA-1* and *Klf1* in *ASC1*-knockdown K562 cells. The data are presented as the relative level to the cells with scrambled shRNA. * p < 0.01 (n = 3). **d.** GATA-1 and KLF1 protein level in ASC1 knockdown cells. **e**. Subcellular localization of ASC1 in UFBP1 and Uba5 knockdown K562 cells. Subcellular fractionation of K562 cells was performed according to [6]. Lamin B1 was used as the nuclear marker, while GAPDH was used as the cytosolic marker. **f.** ChIP analysis of ASC1 association to the promoters of *GATA-1* and *Klf1* genes in BM cells. BM and spleen cells were isolated from phenylhydrazine-treated control and *UFBP1* CKO mice, and subjected to ChIP assays. c-Myc and CCND1 promoters were used as positive ASC1 targets while actin promoter was the negative control. Data are presented as means ± SD (n = 3). **g.** A working model for the role of ufmylation in regulation of hematopoiesis.

ASC1, a co-activator of hormone receptors, is a newly identified Ufm1 target [8]. Its ufmylation promotes recruitment of other transcription factors and thereby enhances estrogen receptor-mediated transcription [8]. Interestingly, in contrast to Uba5 and UFBP1, depletion of ASC1 in K562 cells did not promote *Xbp-1* mRNA splicing and Grp78 up-regulation, indicating that ASC1 knockdown does not cause elevation of basal ER stress (Fig 8A and 8B). However, ASC1 knockdown led to significant under-expression of GATA-1 and KLF1, suggesting a crucial role of ASC1 in regulation of erythroid transcription program (Fig 8C and 8D). We further examined the effect of UFBP1 and Uba5 knockdown on ASC1. Knockdown of either UFBP1 or Uba5 in K562 cells did not change ASC1 protein level (Fig 8E), but caused the reduction of its nuclear presence (Fig 8E). Given that ASC1 functions as a transcription co-activator, we speculated a direct role of ASC1 in transcriptional regulation of erythroid genes such as *Gata-1* and *Klf1*. To test this possibility, we performed chromatin-immunoprecipitation (ChIP) assay using UFBP1-depleted BM and spleen cells to examine whether ASC1 is associated with *Gata-1* and *Klf1* promoters. Depletion of UFBP1 did not alter ASC1 expression in *UFBP1* deficient bone marrow cells (S5A Fig). Both *c-Myc* and *CCND1* (cyclin D1) were reported to be ASC1 targets in breast cancer cells [8]. In agreement with the previous report, ASC1 was indeed associated with the promoters of *c-Myc* and *CCND1*, and more importantly, the associations were significantly decreased in *UFBP1* deficient BM cells (Fig 8F). As a negative control, the beta-actin promoter sequence was slightly enriched in ASC1-DNA complex but the enrichment was not affected by UFBP1 depletion (Fig 8F). Intriguingly, both *Gata-1* and *Klf1* promoter sequences were substantially enriched in ASC1-DNA complex, and the enrichment was significantly diminished by *UFBP1* deficiency (Fig 8F). Similar result was obtained using *UFBP1* deficient spleen cells (S5B Fig). Together, our results strongly suggest that ASC1 is one of the key downstream effectors of the Ufm1 system to transcriptionally regulate erythroid lineage development.

## Discussion

In this study, we report that UFBP1, a key component of the Ufm1 conjugation system, is essential for murine erythropoiesis in both embryonic and adult stages. Germ-line inactivation of *UFBP1* caused anemia and embryonic lethality around E12.5 (Figs 1 and 2), whereas induced ablation of *UFBP1* in adult mice impaired hematopoiesis, resulting in severe pancytopenia and animal death (Figs 3 and 4). We found that defective hematopoiesis in *UFBP1* deficient mice was in part caused by sustained ER stress-induced cell death of HSCs (Figs 5 and 6). Furthermore, ablation of ufmylation capacity, including depletion of Uba5 (E1), UFBP1 (E3 co-activator) or ASC1 (Ufm1 target), significantly down-regulated expression of erythroid transcription factors (Figs 7 and 8). Interestingly, depletion of Ufm1 target ASC1 caused under-expression of *GATA-1* and *Klf1*, but not elevation of ER stress (Fig 7). Taken together, our findings strongly suggest that the Ufm1 system plays crucial pleiotropic roles in regulating both cell survival and differentiation during hematopoiesis. Accordingly, we propose a working model for ufmylation-mediated regulation of hematopoiesis: its activity in maintaining ER homeostasis is essential for survival of hematopoietic stem and progenitor cells, whereas ufmylation of ASC1 is crucial for high-level expression of erythroid transcription factors and erythroid lineage development (Fig 8G)

Previous studies have shown that UFBP1 is a putative Ufm1 target, and its ufmylation is promoted by Ufm1 E3 ligase Ufl1 [2]. Furthermore, Ufm1-modified UFBP1 is required for ufmylation of another Ufm1 target ASC1 [8], indicating that UFBP1 may function as a co-factor of Ufm1 E3 ligase. Interestingly, we found that *UFBP1* KO mice shared extensive phenotypic similarities with *Ufl1* and *Uba5* KO mice in hematopoietic system. First, ablation of either gene impaired embryonic erythropoiesis and caused embryonic lethality around E10.5-E12.5, even though death of *UFBP1* null embryos appeared to occur slightly delayed compared to *Ufl1* null embryos [4, 9]. This delayed death of *UFBP1* null embryos may be attributed to the fact that Ufl1 also regulates C53 protein that is essential for embryogenesis (Fig 1 and [6, 12, 13]). Second, a high number of multi-nucleated erythrocytes were found is all KO embryos (Fig 2 and [4, 9]). Third, *UFBP1* and *Ufl1* KO mice exhibited nearly identical defects in erythroid development (Fig 4 and [9]). Together, these phenotypic similarities strongly suggest that UFBP1 is indeed a key downstream effector of the Ufm1 system in ufmylation-mediated regulation of hematopoietic development. Interestingly, a genome-wide association study (GWAS) study has identified one SNP (rs11697186) in DDRGK1 (same as UFBP1) gene that is strongly associated with anemia and thrombocytopenia in peggylated interferon and ribavirin therapy for chronic hepatitis C patients, which provides clinical evidence for possible involvement of UFBP1 in regulation of erythropoiesis [14]. Nevertheless, it is also worth noting that *UFBP1* deficient mice do have a subtle phenotypic difference compared to *Ufl1* KO mice. Autophagic flux appeared to be normal in *UFBP1* deficient cells (S4 Fig), whereas *Ufl1* deficiency caused inhibition of autophagic degradation [9]. It has been shown that Ufl1 knockdown results in significant depletion of both C53 and UFBP1 proteins [6, 12]. Our result indicates that depletion of UFBP1 protein may not be the causative factor for blockage of autophagic flux in *Ufl1* deficient cells.

Similar to *Ufl1* deficient cells, UFBP1-depeted LSK cells displayed elevated ER stress that in turn caused activation of the UPR and cell death program (Fig 6). ER homeostasis is crucial for survival and function of HSC and other progenitor cells [15]. The UPR is a highly coordinated program that facilitates protein folding, processing, export and degradation of proteins during cellular response to ER stress. It generally includes three signaling branches: IRE1 (Inositol requiring enzyme 1), PERK (PKR-like ER kinase) and ATF6 [16]. Although the UPR serves as a cytoprotective role to restore ER homeostasis, persistent activation of IRE1 and PERK signaling caused by unresolved ER stress also leads to cell death and tissue damage [16]. It has been reported that HSCs and other progenitor cells exhibit distinct cellular response to extracellular ER stressors, and the PERK-eIF2α-CHOP pathway predisposes human HSCs to ER stress-induced apoptosis [15]. Furthermore, overexpression of ER chaperone ERdj4 and RNA-binding protein Dppa5/Esg1 in HSCs reduced ER stress and thereby improved HSC survival and stemness function [15, 17]. Interestingly, we found that depletion of either UFBP1 or Uba5 led to elevated ER stress and activation of the UPR, and sustained ER stress may contribute to cell death of HSCs (Figs 6 and 7). This result is line with the previous studies showing that the Ufm1 system protects pancreatic beta cells and macrophages from ER stress-induced apoptosis [7, 11]. Our finding suggests that the Ufm1 system plays a cytoprotective role by maintaining ER homeostasis of HSCs. Therefore, manipulation of ufmylation activity may represent a novel approach to improve survival and function of HSCs.

In addition to their role in maintaining ER homeostasis that appears to be a general function in most types of cells we examined, UFBP1 and Ufl1 also possess a lineage-specific activity, which was evidenced by the complete blockage of differentiation of CFU-Es to proerythroblasts in both *UFBP1* and *Ufl1* deficient bone morrows (Fig 4A and [9]). This lineage-specific effect was also observed in *Uba5* deficient embryos, in which development of MEP (megakaryocyte erythroid progenitors), but not GMPs (granulocyte macrophage progenitors), was impaired in fetal livers [4]. Yet, the molecular basis of this erythroid-specific activity remains largely unknown. In this study, we found that ASC1, a transcription co-activator and Ufm1 target, was the key downstream effector to mediate this lineage-specific activity. ASC1 was originally identified as thyroid hormone receptor interactor 4 (TRIP4) [18, 19]. As a transcriptional co-activator, ASC1 enhances transcription by recruiting nuclear receptors (such as ERα), transcriptional cofactors (p300 and SRC1), and basic transcriptional machinery (TBP and TFIIA) to specific promoters, and its ufmylation provides a platform for the recruitment of these factors [8]. Interestingly, it has been known that nuclear receptor signaling pathways play a crucial role in both normal and stress erythropoiesis. Altering the content of thyroid hormone receptor α (TRα) in chicken erythroblasts affects the balance between proliferation and differentiation [20]. *TRα* ^-/-^ mice display defective fetal and adult erythropoiesis and an altered stress erythropoiesis response to hemolytic anemia [21, 22]. Moreover, glucocorticoid receptor (GR) signaling is important for stress erythropoiesis [23], while retinoic acid (RA) has a broad impact on hematopoiesis [24]. Estrogen receptor was also reported to modulate HSC survival and erythropoiesis [25]. Therefore, it is quite plausible that ASC1 and its ufmylation may be directly involved in regulation of erythroid development. To support our hypothesis, we found that knockdown of ASC1 in K562 down-regulated key erythroid transcription factors GATA-1 and KLF1 (Fig 8C). More importantly, ChIP analysis showed that ASC1 was associated with the promoters of *GATA-1* and *Klf1*, and this association was significantly attenuated by depletion of UFBP1 (Fig 8E), suggesting that ufmylation of ASC1 is important for its association with the promoters of *GATA-1* and *Klf1*. Further studies are needed to identify and characterize both extracellular and intracellular signals leading to ASC1 ufmylation and its binding partners, and to determine the molecular mechanism of ASC1-mediated regulation of erythroid development. In addition, our results also indicate an important role of UFBP1 in development of other lineages such as myeloid cells. Tissue- and lineage-specific genetic models will be generated for thorough elucidation of its function and working mechanism in the hematopoietic system.

## Materials and Methods

### Ethics statement

Our animal study was conducted according to the guidelines of Animal Research: Reporting In Vivo Experiments (ARRIVE). IACUC of Georgia Regents University approved this study (protocol #2011–0314)

### Tissue culture cells and chemical reagents

K562 cells were cultured in RPMI 1640 medium supplemented with 10% FBS and antibiotics. Tamoxifen and 4-hydroxytamoxifen and other chemical reagents were purchased from Sigma (St. Louis, MO, USA).

### Generation of UFBP1 KO and CKO mice and genotyping

ES cell clone HEPD0618_2_D02 (JM8A3.N1 with C57BL/6N background) containing trapped *UFBP1* allele was purchased from the EUCOMM (European Conditional Mouse Mutagenesis) team. The ES cells were injected into the blastocysts of C57BL/6 mice (Northwestern University Transgenic and Targeted Mutagenesis Laboratory). Chimeric mice were crossed with B6(Cg)-Tyr^*C-2J*^/J albino mice, and heterozygous offspring with germ-line transmission were confirmed by genotyping.

To generate CKO mice, we crossed *UFBP1* ^Trap-F/+^ mice with FLPo deleter mice (B6(C3)-Tg(Pgk1-FLPo)10Sykr/J, The Jackson Laboratory, Bar harbor, ME) to remove the gene trap cassette. The floxed *UFBP1* mice were crossed with ROSA26-CreERT2 mice (B6.129-Gt(ROSA)26Sor<tm1(cre/ERT2) Tyj>/J, The Jackson Laboratory) in which CreERT2 was inserted into ROSA26 locus. Cre-mediated deletion of *UFBP1* was induced by tamoxifen administration. Tamoxifen (20 mg/ml in corn oil, Sigma, St. Louis, MO) was administrated by 5-day IP injection with an approximate dose of 75 mg tamoxifen/kg body weight. All animal procedures were approved by IACUC of Georgia Regents University.

The following primers were used for PCR genotyping of *UFBP1* KO embryos and mice: P1 (TAGTACTTGAAGTCTGGCTTGGTA), P2 (CACAACGGGTTCTTCTGTTAGT CC) and P3: (TAGTCAGGAACTGATGA GTGTCTC). A 35-cycle (92°C, 45 sec, 62°C, 45 sec, 72°C 45 sec) PCR was performed in the present of 1% DMSO, and the determination of genotypes was described in Fig 1. For the floxed *UFBP1* allele, P1 and P3 primers were used in PCR genotyping. The floxed allele generates a 637 bp PCR product comparing to 440 bp WT product. Genotyping of Cre-ERT2 mice was performed according to the standard protocol of the Jackson Laboratory.

### Complete blood count and colony formation assays

Fetal liver cells (5,000 cells) were plated in one ml of serum-free methylcellulose and IMDM (M3234, Stem Cell Technology, Vancouver, BC, Canada) supplemented with 10% fetal bovine serum (FBS). For CFU-Es, cells were cultured for 3 days in the presence of hEpo (2 units/ml, Epogen, Amgen, Thousand Oaks, CA) and stained with benzidine dihydrochloride, and the number of colonies is scored. For BFU-Es, cells were cultured in the presence of hEpo (2 units/ml) and mSCF (100 ng/ml, Peprotech, Rocky Hill, NJ) for 7 days, stained and scored. For CFU-GMs, CFU-GEMMs, BM or fetal liver cells (20,000) were cultured in M3434 (Stem Cell Technology) for 5 days, and the numbers of the colonies were manually counted. For EryP-CFCs, yolk sac cells from E8.5 embryos was cultured in IMDM medium containing 10% plasma-derived serum, 5% PFHM-II, 0.025 mg/ml ascorbic acid, 4.5 x 10^−4^ M MTG, Epo (2 U/ml), and colonies were manually counted after 96 hours.

Complete blood counts (CBC) of blood samples from adult mice were analyzed by in-house Horiba ABX Micro S60 analyzer (HORIBA Medical, Irvine, CA, USA).

### Competitive repopulation assay

Recipient mice (CD45.1) were first irradiated with dose of 9 Gy. BM cells from *UFBP1* ^F/F^ and *UFBP1* ^F/F^;ROSA-CreERT2 mice (CD45.2) were isolated, mixed with BM cells from CD45.1 mice in a 1:1 ratio (1x10^6^ cells total), and subsequently injected into the retro-orbital venous sinus of anesthetized recipient mice (5 mice for each group). After a 4-week recovery period, tamoxifen was administered in 5 consecutive days. Donor cell engraftment was monitored by flow cytometry of the blood samples from tail veins in a 2-week interval using lineage markers. After 3 weeks of tamoxifen injection, BM cells were subjected to flow analysis.

### In vitro culture of LSK progenitor cells

Sorted BM HSC and myeloerythroid progenitor cells were cultured in IMDM supplemented with 10% fetal bovine serum, hEpo (2 units/ml, Amgen, Thousand Oaks, CA), mSCF (100 ng/ml, Peprotech, Rocky Hill, NJ), mIL-3 (10 ng/ml, Peprotech), Dexamethasone (1 μM, Sigma, St. Louis, MO, USA) and IGF-1 (100 ng/ml, Invitrogen, Grand Island, NY). Deletion of *UFBP1* was induced by addition of 4-hydroxytamoxifen (1 μM, Sigma).

### Flow cytometry analysis and cell sorting

Freshly isolated BM cells were suspended in PBS with 1% fetal bovine serum and stained with BV510-Sca-1 (Biolegend, San Diego, CA), APC780-c-Kit (eBioscience, San Diego, CA), PE-Cy7-CD150 (Biolegend), Alexa700-CD16/32 (eBioscience), PE-IL-7R (BD Biosciences), APC-CD41 (BD Biosciences), BV650-CD105 (Biolegend), FITC-CD71 (BD Biosciences), BV421-TER119 (Biolegend), and PerCP-Cy5.5 conjugated lineage markers including CD4, CD8, CD3, CD5, Gr-1, CD11b, CD19 and B220 (BD Biosciences). The samples were analyzed using BD LSR II SORP and Diva 7.0 software (GRU Cancer Center Flow Cytometry Facility). For competitive repopulation assays, BV421-CD45.2 (Biolegend) was used to substitute BV421-TER119. Cell sorting of HSCs and progenitor cells was performed using BD FACSAria II SORP.

### Quantitative real-time PCR (qRT-PCR)

Total RNA from each sample was isolated with the GeneJET RNA Purification Kit (Thermo Scientific), and then reversely transcribed using the SuperScript First-Strand Synthesis System (Invitrogen) according to the manufacturer’s instruction. RT-PCR was performed using the iTaq Universal SYBR Green Supermix kit (BIO-RAD) with 40 cycles of 95°C for 15 seconds and 60°C for 1 minute on StepOnePlus Real-Time PCR System (Life Technologies). The results were analyzed by StepOne Software (Version 2.1, Life Technologies). Relative expression level of each transcript was normalized to murine beta-actin and GAPDH by using the 2^(-delta delta Ct) method. The following is the list of primers used in this study:

Genes Primer sequence

Mouse Actin     GACCTCTATGCCAACACAGT

                 AGTACTTGCGCTCAGGAGGA

Mouse UFBP-1    GAAGCCAGCAGAAGTTCACC

                 GAAGCCGTTCCTCTTCCTTC

Mouse Grp78     ACTTGGGGACCACCTATTCCT

                 ATCGCCAATCAGACGCTCC

Mouse ERdj4     TAAAAGCCCTGATGCTGAAGC

                 TCCGACTATTGGCATCCGA

Mouse CHOP      GCATGAAGGAGAAGGAGCAG

                 ATGGTGCTGGGTACACTTCC

Mouse GADD34    AGAACATCAAGCCACGGAAG

                 TCTCAGGTCCTCCTTCCTCA

Mouse Bax       TGCAGAGGATGATTGCTGAC

                 AAGATGCTGTTGGGTTCCAG

Mouse Bak       AAAATGGCATCTGGACAAGG

                 AAGATGCTGTTGGGTTCCAG

Mouse Bim       TGCAGAGGATGATTGCTGAC

                 GATCAGCTCGGGCACTTTAG

Mouse Noxa      GGCAGAGCTACCACCTGAGT

                 TTGAGCACACTCGTCCTTCA

Mouse Puma      GCCCAGCAGCACTTAGAGTC

                 TGTCGATGCTGCTCTTCTTG

Mouse DR5       TGACTACACCAGCCATTCCA

                 AGTTCCTCTTCCCCGTCAGT

Human Actin     CCTGTACGCCAACACAGTGC

                 ATACTCCTGCTTGCTGATCC

Human Gata1     GATGGAATCCAGACGAGGAA

                 GCCCTGACAGTACCACAGGT

Human Klf1      CACGCACACGGGAGAGAAG

                 CGTCAGTTCGTCTGAGCGAG

Human UFBP-1    GAAGCCAGCAGAAGTTCACC

                 GAAGCCGTTCCTCTTCCTTC

Human Grp78     ACTTGGGGACCACCTATTCCT

                 ATCGCCAATCAGACGCTCC

Human ASC1      AGTGGGTTGACCACACAGGT

                 CTGCCCTCCACCCTTTTAAT

### Chromatin immunoprecipitation (ChIP) assay

For ASC1 ChIP assay, *UFBP1* ^F/F^ (control) and *UFBP1* ^F/F^;CreERT2 mice were injected with tamoxifen for 5 consecutive days. At day 4, mice were injected with a single dose of phenylhydrazine (40 mg/kg). Bone marrow and spleen cells were harvested at day 8, and subjected to ChIP and quantitative PCR analysis as previously described [26, 27]. The following primers were used for detection of specific promoters: mKLF1-chip, AGCGACCAGGACTTTTCTTT (F) and CGATATGTGTGT GGTGTGCT (R); mGATA1-chip, CCAACTCCTGCAAGAACA GT (F) and GTTGACACTA GCCCCACAGT (R). mActin-chip, TCTCCAAAAGTGCCTGACTC (F) and CCCTTCTGCTGTGGTTCTAA (R); mMyc-chip, CGACCAAAGGCAAAATACAC (F) and CCCTGCGTATATCAGTCACC (R); mCCND1-chip, CTCCCCCAACTCAAGACTG (F) and AATTCCAGCAACAGCTCAAG (R).

### Xbp-1 mRNA splicing assay

*Xbp-1* mRNA splicing assay was conducted as described previously [26], using the following primers: for human *Xbp-1*: 5’ GGAGTTAAGACAGCGC TTGG 3’ (F) and 5’ ACTGGGTCCAAGTTGTCCAG 3’ (R); and for murine *Xbp-1*, 5’ ACACGCTTG GGAAT GACAC 3’ (F) and 5’ CCATGGGAAGATGTTCTGGG 3’ (R).

### shRNA constructs

Lentiviral vectors expressing specific shRNAs were constructed using pLKO.1 vector. Lentiviruses were prepared using 293FT packaging cell line according to the manufacturer's instruction (Invitrogen). The following shRNAs were used:

hUba5 shRNA:       CCTCAGTGTGATGACAGAAAT;

hUFBP1 shRNA#1: GTGTCGAGAAGCCAGCGGAAA;

hUFBP1 shRNA#2: GGAGCTAAGAAACTGCGGAAG;

hASC1 shRNA#1:   GCAGAGTATCATAGCAGACTA;

hASC1 shRNA#2:   GGACTAGAGTTCAACTCATTT.

For knockdown assays, K562 cells were infected with lentiviruses expressing either scrambled or gene-specific shRNAs for 24 hours, and then selected with puromycin (1.5 μg/ml) and culture for 3 to 4 days. Knockdown efficiency was evaluated by either immunoblotting or quantitative RT-PCR.

### Antibodies, immunoblotting and immunofluorescence staining

Immunofluorescent staining and Immunoblottings were performed as described previously^6^. Confocal images were acquired using Zeiss 510 META confocal microscope with Zen software (Carl Zeiss Microscopy GmbH, Jena, Germany), while epifluorescence images were obtained using Zeiss Observer D1 with AxioVision 4.8 software (Carl Zeiss Microscopy GmbH, Jena, Germany).

The antibodies used in this study include: UFBP1 and Uba5 rat antibody (Li lab and [6, 26]), eIF2α, phospho-S51-eIF2α (Cell Signaling, Danvers, MA, USA), ASC1 (A300-843A, Bethyl Laboratories, Montgomery, TX, USA), JNK and phospho-JNK (Santa Cruz Biotechnology Inc., Dallas, TX, USA), GATA-1 and KLF1 (Abcam, Cambridge, MA, USA), α-tubulin and β-actin (Sigma). All affinity-purified and species-specific HRP- and fluorophore-conjugated secondary antibodies were obtained from Jackson ImmunoResearch (West Grove, PA, USA).

### TUNEL staining

TUNEL staining was performed using In situ cell death detection kit (Fluorescein, Roche, Indianapolis, IN, USA) according to the manufacturer’s instruction.

### Histology

For hematoxylin and eosin (H & E) staining of embryo sections, the embryos were fixed in 10% formalin overnight, rinsed with PBS and embedded in paraffin. Embryo sections were deparaffinized and hydrated, and subsequently stained with hematoxylin solution for 6 minutes. After rinse in water for 15 minutes, the sections were stained with eosin-Y solution for 3 minutes. The sections were then rinsed in water, dehydrated and mounted with Permount.

## Supporting Information

S1 Fig Representative photographs of wild-type and *UFBP1* KO embryos (E11.5). **a.** Embryos with yolk sac; and **b.** Embryos without yolk sac.
(TIF)

S2 Fig Survival curve of lethally irradiated wild-type mice after transplantation of TAM-treated BM cells from either *UFBP1* ^F/F^ or *UFBP1* ^F/F^:CreERT2 mice. p < 0.002 (n = 5 each group). The unfractionated BM cells from either *UFBP1* ^F/F^ or *UFBP1* ^F/F^:CreERT2 (CD45.2) were transplanted into lethally irradiated recipient CD45.1 mice.
(TIF)

S3 Fig Contribution of *UFBP1* deficient cells (CD45.2) to L^-^S^+^K^+^ cells in competitive repopulation assay. * p < 0.001 (n = 5). The experimental procedure was described in the legend of Fig 5.
(TIF)

S4 Fig Protein level of LC3B and p62 in UFBP1-depleted BM cells. After 3-week treatment of TAM, the total BM cells were collected and the cell lysates were subjected to immunoblotting of specific antibodies.
(TIF)

S5 Fig a. Protein level of ASC1 in UFBP1-depleted BM and spleen cells. b. ChIP analysis of ASC1 association to the promoters of *GATA-1* and *Klf1* genes in spleen cells. Spleen cells were isolated from phenylhydrazine-treated control and *UFBP1* CKO mice, and subjected to ChIP assays. c-Myc and CCND1 promoters were used as positive ASC1 targets while actin promoter was the negative control. Data are presented as means ± SD (n = 3).
(TIF)
