# Supplementary figures and images for: UFBP1, a Key Component of the Ufm1 Conjugation System, Is Essential for Ufmylation-Mediated Regulation of Erythroid Development
Source: PLoS Genet. 2015 Nov 6;11(11):e1005643. doi: 10.1371/journal.pgen.1005643 (PMC4636156; doi:10.1371/journal.pgen.1005643)

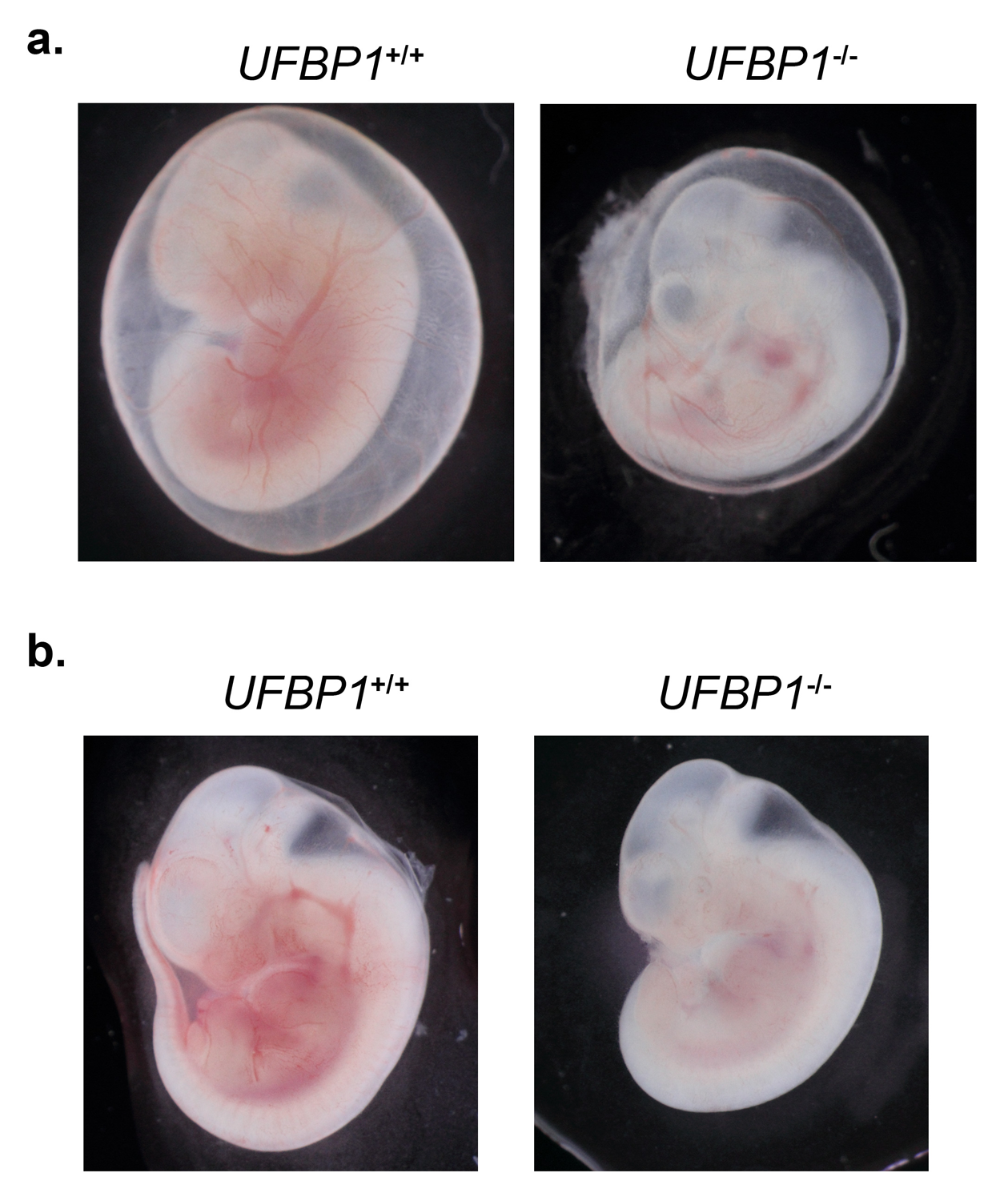

Supplement: S1 Fig — a. Embryos with yolk sac; and b. Embryos without yolk sac. (TIF) [file pgen.1005643.s001.tif]

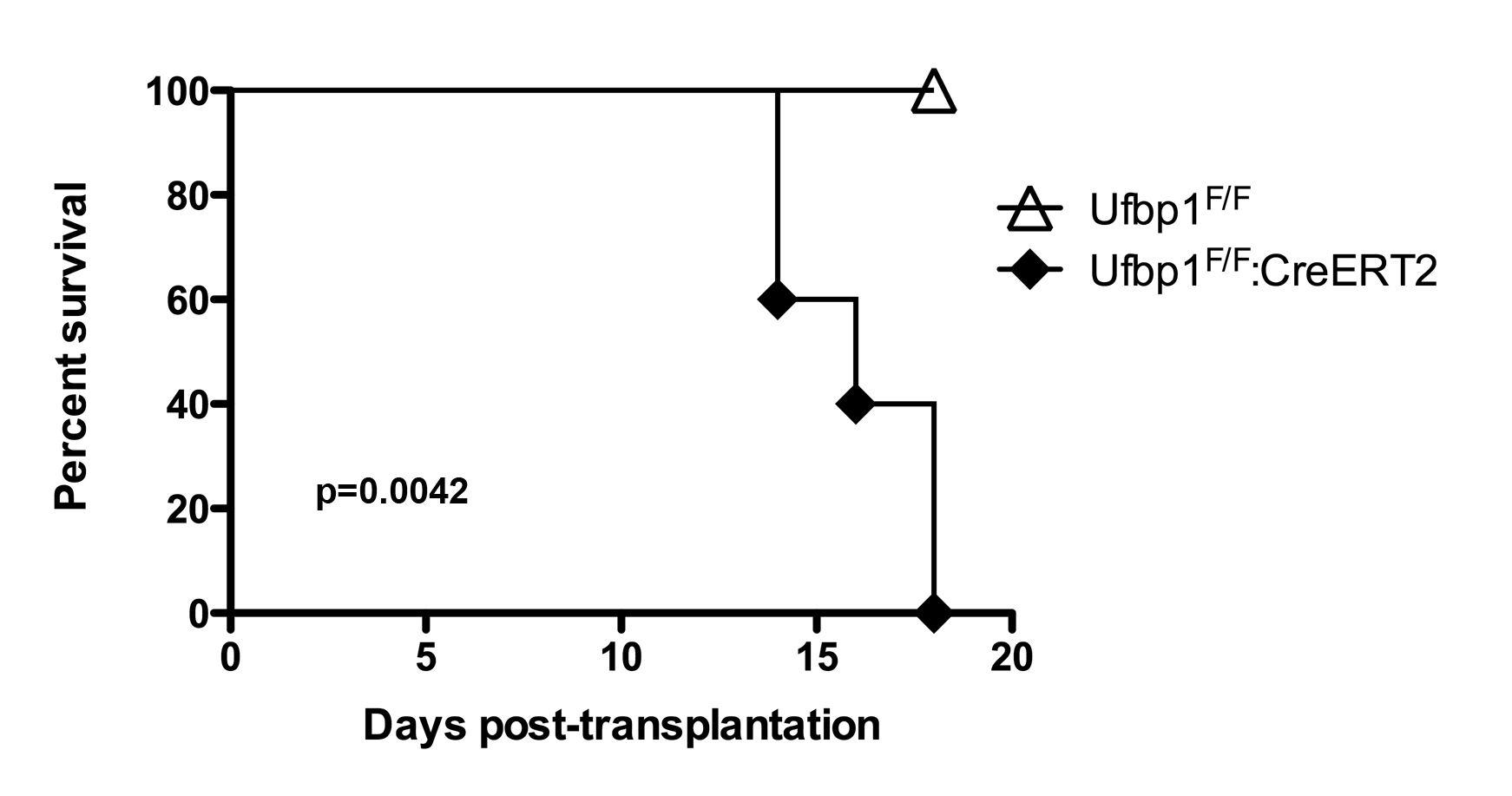

Supplement: S2 Fig — The unfractionated BM cells from either UFBP1 F/F or UFBP1 F/F:CreERT2 (CD45.2) were transplanted into lethally irradiated recipient CD45.1 mice. (TIF) [file pgen.1005643.s002.tif]

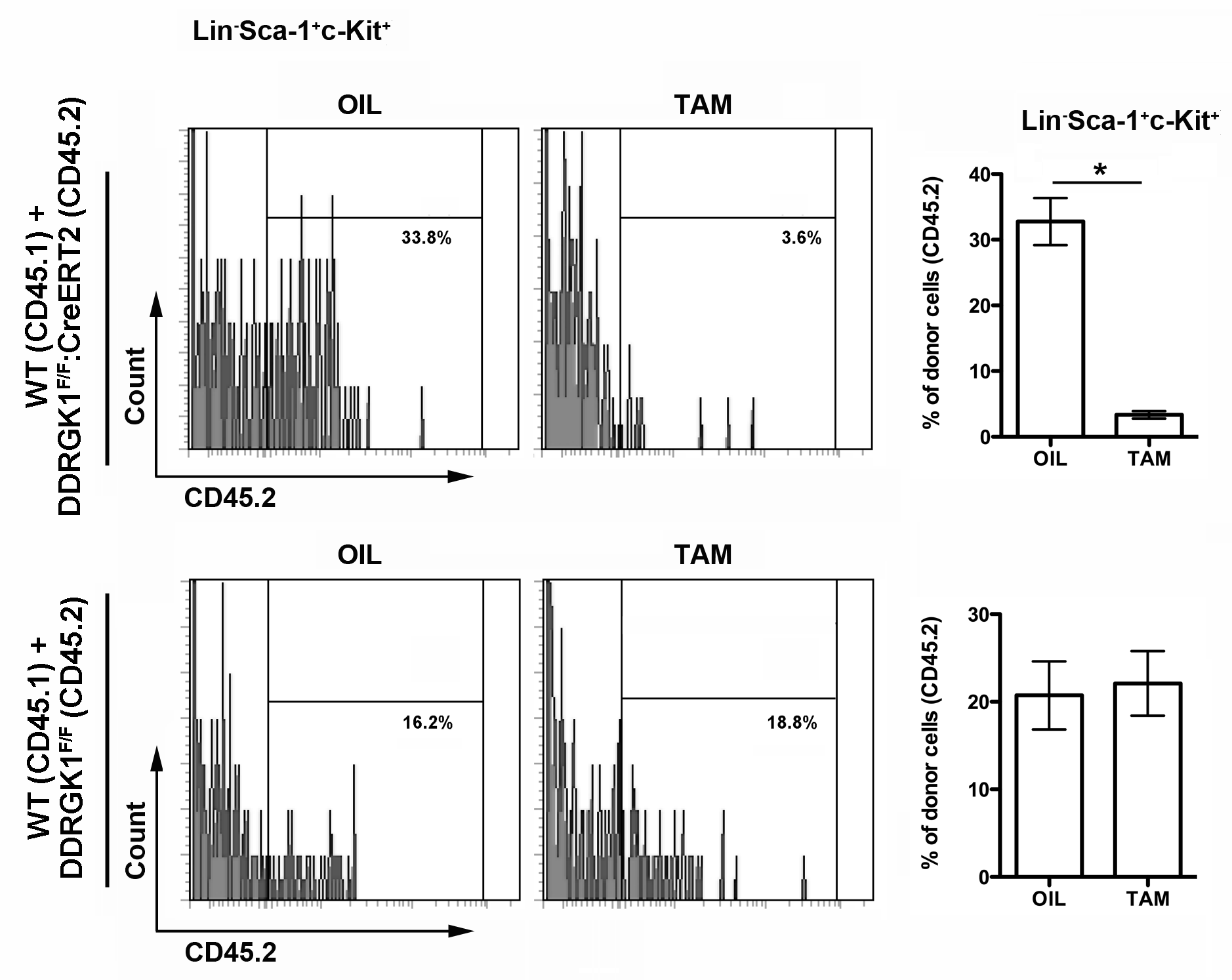

Supplement: S3 Fig — * p < 0.001 (n = 5). The experimental procedure was described in the legend of Fig 5. (TIF) [file pgen.1005643.s003.tif]

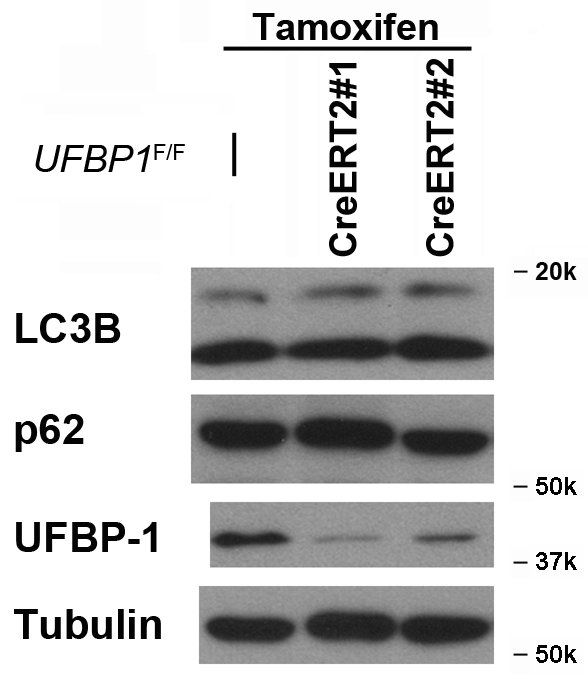

Supplement: S4 Fig — After 3-week treatment of TAM, the total BM cells were collected and the cell lysates were subjected to immunoblotting of specific antibodies. (TIF) [file pgen.1005643.s004.tif]

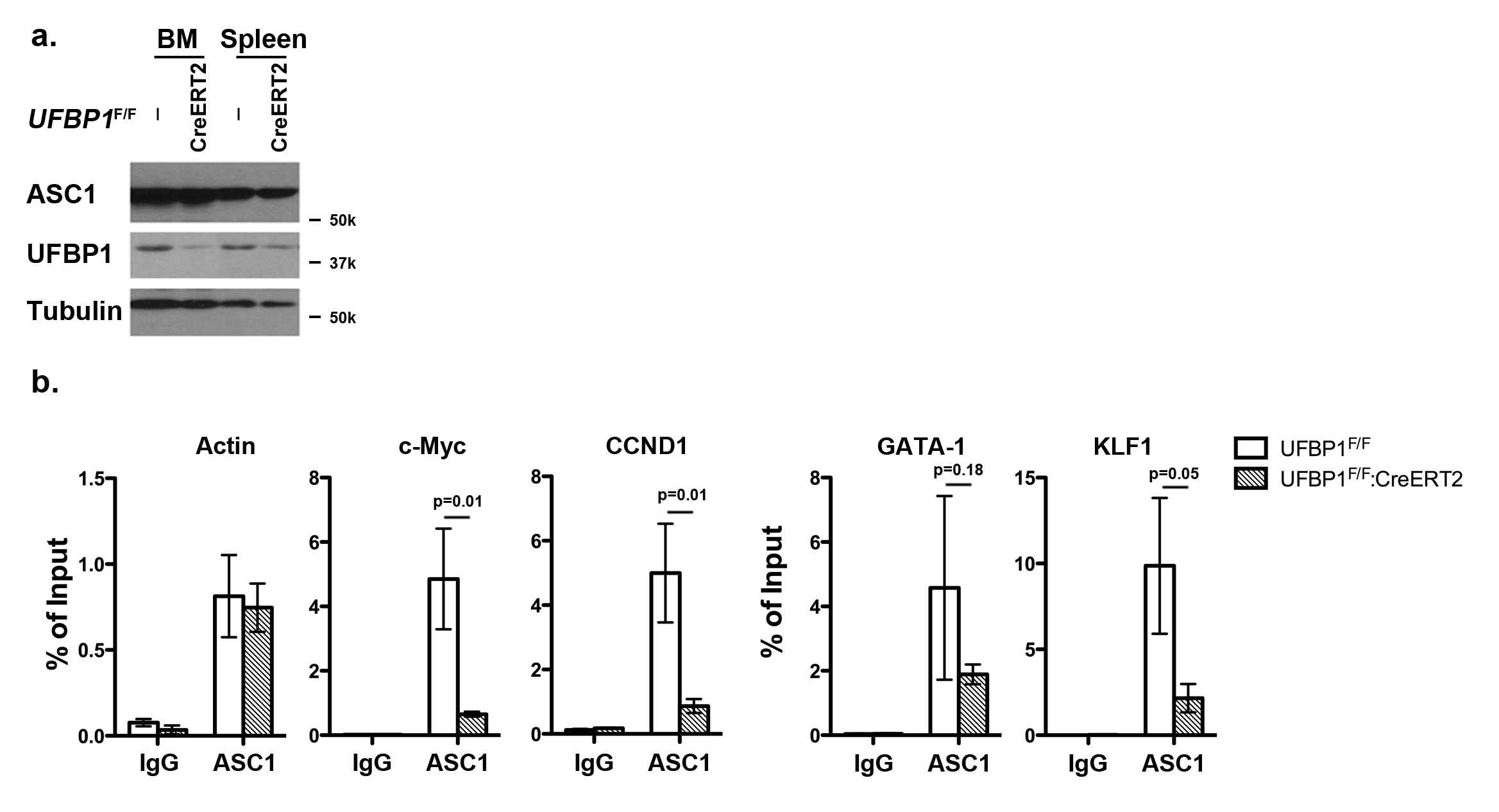

Supplement: S5 Fig — b. ChIP analysis of ASC1 association to the promoters of GATA-1 and Klf1 genes in spleen cells. Spleen cells were isolated from phenylhydrazine-treated control and UFBP1 CKO mice, and subjected to ChIP assays. c-Myc and CCND1 promoters were used as positive ASC1 targets while actin promoter was the negative control. Data are presented as means ± SD (n = 3). (TIF) [file pgen.1005643.s005.tif]
